# Consequences of the 2022 Intravenous Contrast Shortage on Emergency Department Care: A Retrospective Study

**DOI:** 10.5811/westjem.62947

**Published:** 2026-05-12

**Authors:** Shawna Bellew, Lindsay Tjiattas-Saleski, Daniel Butz, Mandy Stallard, Matt Galush, Nathan Hudepohl, Michael Ramsay, Sabrina Avanzato, Constantine Hrysikos, Riley Seay

**Affiliations:** Edward Via College of Osteopathic Medicine, Carolinas Campus, Department of Emergency Medicine, Spartanburg, South Carolina

## Abstract

**Introduction:**

Computed tomography (CT) is an essential diagnostic tool for evaluating emergency department (ED) patients. Between 15–20% of patients who present to the ED in the United States (US) undergo CT imaging. CT imaging with the use of intravenous contrast media (ICM is used in the evaluation of abdominal pain, concern for active bleeding, vascular pathology, and pulmonary embolism (PE). In April of 2022, production shut-downs in Shanghai, China due to the COVID-19 pandemic resulted in a global shortage of ICM.

**Objective:**

To examine the impact of the ICM shortage due to the COVID-19 pandemic on patient outcomes and ED operations.

**Methods:**

We performed a retrospective study of adult patients (age 18 and older) who received either CT imaging at six Prisma Health EDs before (June to July 2019) and during (June to July 2022) the contrast shortage. Data was electronically extracted from the electronic medical record. Main outcomes included 30-day mortality, 30-day return-visit, and ED length of stay. We used the t-Test, paired t-Test, or ANOVA for normally distributed data and logistic regression to assess the likelihood of undergoing a CT with ICM.

**Results:**

We analyzed 11,044 patients who received CT imaging. ICM was used in 93% of CT scans during the non-shortage period and 45% during the shortage. (p < 0.001). The likelihood of a return visit within 30 days decreased during the ICM shortage period and the non-shortage period by 6.0% and 4.3%, respectively (Fisher’s Exact Test, p-value = 6.71e-05). The shortage did not have a statistically significant effect on patient mortality within 30 days of ED visit staying stable at 2.8% (Fisher’s Exact Test p-value ≈ 0.89). There was a statistically significant increase in patient time-first-roomed upon ED arrival. (p-value < 0.001). However, there was a statistically significant increase in ED length of stay (LOS, p = 0.04).

**Conclusion:**

The rationing of ICM had did not have a statistically significant effect on 30-day mortality. There was a decrease in 30-day return-visit likelihood which was statistically significant. Despite the reduced ICM usage, there was a statistically significant increase in ED LOS during the shortage period. However, more factors need to be considered as the COVID-19 pandemic could have made an impact on hospital operations. This demonstrates that there may be opportunities to decrease ICM usage without causing negative effects on mortality or morbidity.

